# The mir-675-5p regulates the progression and development of pancreatic cancer via the UBQLN1-ZEB1-mir200 axis

**DOI:** 10.18632/oncotarget.15330

**Published:** 2017-02-15

**Authors:** Jue Wang, Youli Zhang, Hong Wei, Xingxing Zhang, Yan Wu, Aihua Gong, Yu Xia, Wenbing Wang, Min Xu

**Affiliations:** ^1^ Department of Gastroenterology, Affiliated Hospital of Jiangsu University, Jiangsu University, Zhenjiang 212000, China; ^2^ Department of Public Health, School of Medicine, Jiangsu University, Zhenjiang 212000, China; ^3^ Department of Cell Biology, School of Medicine, Jiangsu University, Zhenjiang 212000, China

**Keywords:** Mir-675, mir-200, ZEB1, UBQLN1, pancreatic cancer progression

## Abstract

Pancreatic cancer (PC) is a highly lethal disease due to extensive metastatic lesions. Accumulating evidence suggests that miR-675-5p plays different roles in metastasis through the regulation of epithelial to mesenchymal (EMT) and the mesenchymal to epithelial transitions (MET) in different cancers. ZEB1 promotes the EMT process by controlling the expression of E-cadherin and may have a reciprocal regulation with Ubiquilin1 (UBQLN1) and mir-200 family in cancer progression. In the present study, we showed that decreased expression of miR-675-5p is associated with the enhanced cell proliferation and survival of PC cells, while the increased expression of mir-675-5p shows the opposite one. The mir-675-5p could decrease the expression of mir-200 which is intermediated by ZEB1, and increase the expression of *UBQLN1* gene. The mir-675-5p can increase the expression of ZEB1 mRNA, but the ZEB1 protein level was decreased. When mir-675-5p mimics and siUBQLN1 were co-transfected into the pancreatic cancer Patu8988 cells, the expression of ZEB1 protein was increased. It suggests that mir-675-5p may affect ZEB1 in a post-transcriptional level which was verified to be regulated by UBQLN1 protein. Hence, mir-675-5p regulates the progression of pancreatic cancer cells through the UBQLN1-ZEB1-mir200 pathway.

## INTRODUCTION

Pancreatic ductal adenocarcinoma (PDAC) is now the third most lethal cancer in United States as it has overtaken breast this past year. PDAC is usually diagnosed at advanced stage and is often resistant to therapy [1]. More than 90% of patients die from the disease due to extensive metastatic lesions [2, 3]. Tumor markers such as carcinoembryonic antigen (CEA) and carbohydrate antigen 19-9 (CA19-9) are frequently used in screening for pancreatic cancer, but they lack sensitivity or specificity [4]. Therefore, there is a critical need for better understanding of molecular mechanisms underlying proliferation, apoptosis and metastasis of pancreatic cancer such that we would be able to develop novel prognostic biomarkers and therapeutic targets for the malignancy.

MicroRNAs (miRNAs) are small RNAs with ∼22 nucleotides in length and have been shown to regulate gene expression at the post-transcriptional level, causing translation repression or RNA degradation [5, 6]. miR-675 is embedded within the first exon of long non-coding RNA (lncRNA) H19 [7]. Dysregulation of miR-675 has been reported in a range of embryonic and extra-embryonic cell lines and upregulation of miR-675 can cause reduction of proliferation [8]. Of interest, H19 and miR-675 play different roles in metastasis through the regulation of epithelial to mesenchymal (EMT) and the mesenchymal to epithelial transitions (MET) in different cancers as they can respond to various stress conditions such as reduced p53 and hypoxia, leading to activation of a tumorigenic program of cell survival [9, 10]. Next-generation sequencing suggests that H19 and miR-675 are down-regulated in neoplastic tissue compared to adjacent tissues in pancreatic cancer [11]. This study showed that upregulation of miR-675 inhibited pancreatic cancer cell proliferation and colony formation, and induced the apoptosis *in vitro*. As miR-675-5p has a significant effect on cell proliferation in this study, we select the retinoblastoma (Rb) gene which negatively regulates the G1-S transition by binding to the E2F transcription factors [12].

MicroRNAs can be regulated by a different miRNA through intermediate genes. For example, miR-27b can upregulate miR-508-5p through p53, and this effect is dependent on the negative regulation of p53 by CCNG1 [13].

The miR-200 family is usually considered as tumor suppressor miRNAs and involved in inhibition of EMT [14]. However, Li *et al* reported that miR-200a and miR-200b were hypomethylated and over-expressed in pancreatic cancer compared to adjacent mucosa [15]. ZEB1 is an EMT activator and plays a crucial role in tumor progression towards metastasis. ZEB1 and miR-200 family members repress expression of each other in a reciprocal feedback loop [16]. Our results indicated that over-expression of miR-675-5p could inhibit cell migration and invasion of pancreatic cancer which was closely associated with the EMT related protein ZEB1. We are interested in exploring whether there was a relationship between miR-200 and miR-675-5p by an intermediate gene ZEB1. The mir-675-5p can increase the expression of ZEB1 mRNA, but the ZEB1 protein level was decreased. We supposed that there is a post-transcriptional regulation on ZEB1. Shah *et al* reported that ZEB1 is required for induction of mesenchymal-like properties following loss of UBQLN1 and ZEB1 is capable of repressing expression of UBQLN1, suggesting a physiological, reciprocal regulation of EMT by UBQLN1 and ZEB1 [17].

## RESULTS

### Clinical significance of miR-675-5p in pancreatic cancer

**Figure 1:**
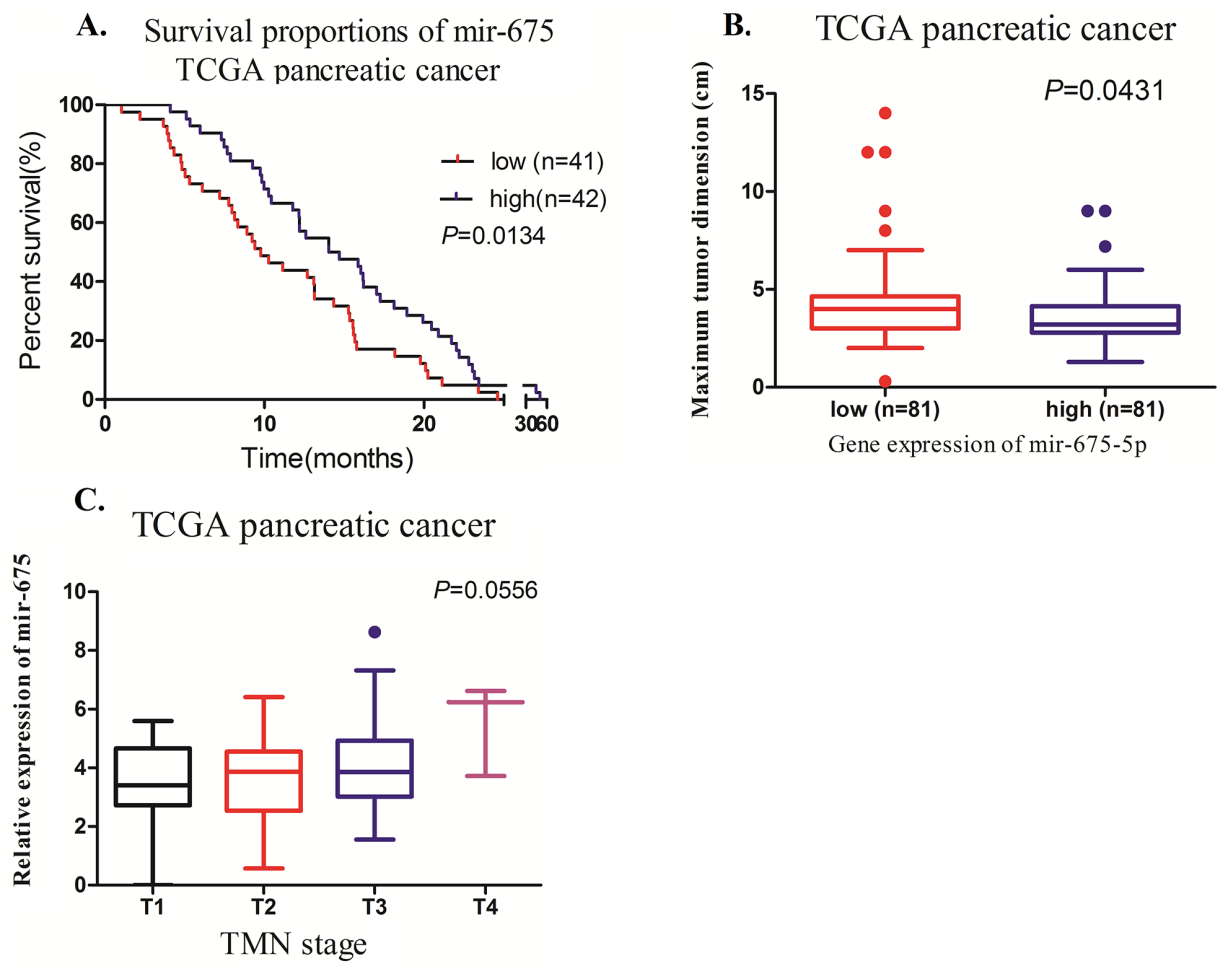
Clinical significance of miR-675-5p in pancreatic cancer from TCGA database **A**. The association between mir-675 expression and the overall survival period of PC patients was analyzed (*P*=0.0134). **B**. The relationship between mir-675 expression and maximum tumor dimension of PC patients was analyzed (*P*=0.0431). **C**. The correlation between mir-675 expression and TMN stage of PC patients was analyzed (*P*=0.0556).

We determined the clinical significance of miR-675-5p by interrogating the TCGA datasets which consist of 14 cancer types through GISTIC2 algorithm (http://www.cbioportal.org/) to identify gene amplifications and mRNA expression in patient tumor samples [18]. We searched and analyzed the TCGA pancreatic cancer related database (196 specimens). Although there was not statistically significant on the relationship between the expression of miR-675-5p and TMN stage, high expression of miR-675-5p had better survival proportions and smaller maximum tumor dimension than low expression of miR-675-5p (Figure 1). This result suggested that miR-675-5p is a tumor suppressor in pancreatic cancer.

### The role of Mir-675-5p in cell proliferation and apoptosis of pancreatic cancer

**Figure 2:**
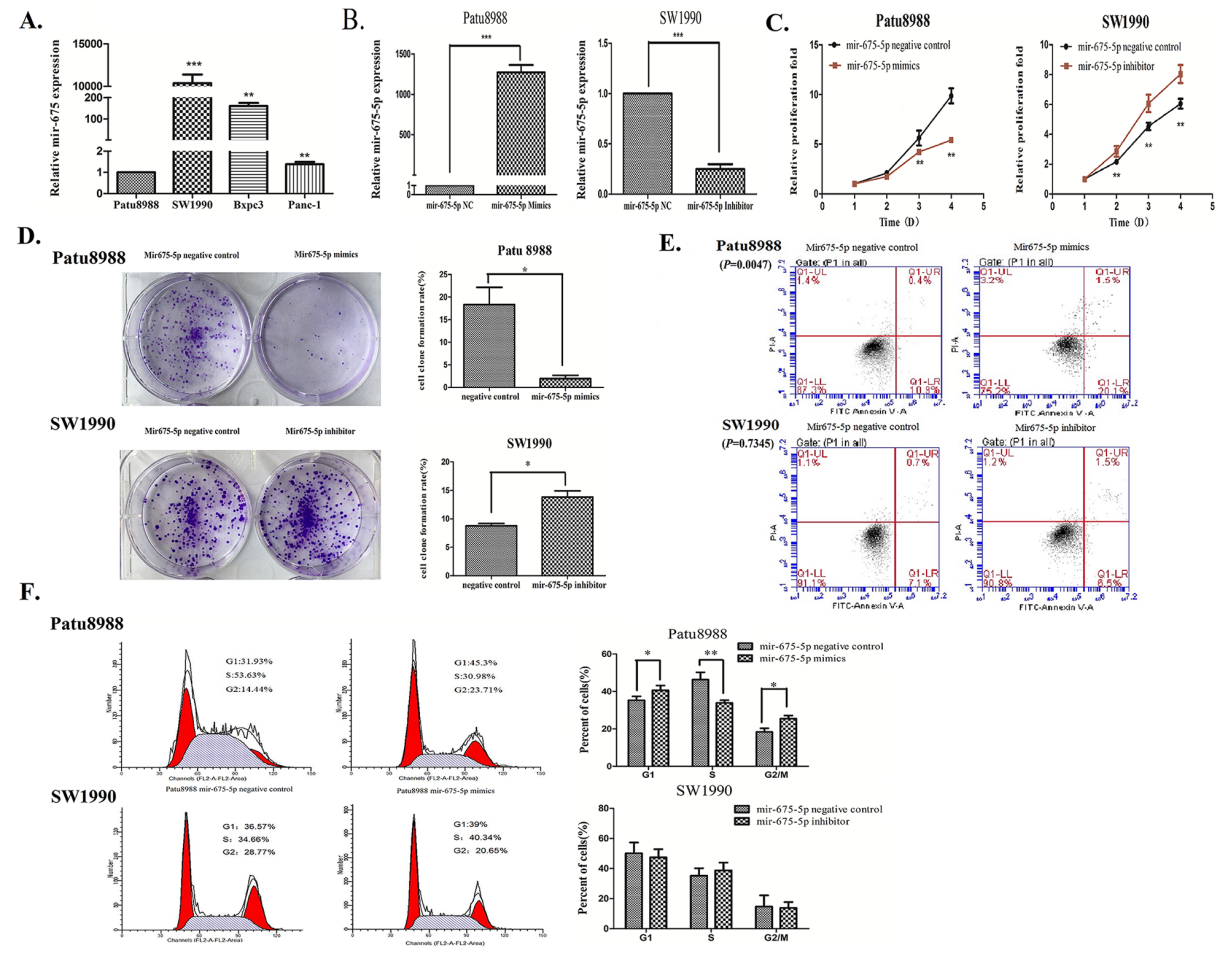
The role of mir-675-5p in cell proliferation and apoptosis of pancreatic cancer **A**. The relative expression of mir-675-5p among four cell lines (Patu8988, SW1990, Bxpc3 and Panc-1) was detected by qRT-PCR. **B**. Mir-675-5p mimics or inhibitors were transfected into Patu8988 and SW1990 cells respectively. The transfection efficiency was measured by qRT-PCR. **C**. CCK-8 assays were conducted to detect the relative proliferation fold of the pancreatic cancer cells. **D**. The colony formation assays were used to examine the ability of proliferation. **E**. Annexin V fluorescein isothiocyanate (V-FITC) apoptotic assays were conducted by flow cytometry to detect the apoptotic level of cells. (Patu8988:*P*=0.0047, SW1990:*P*=0.7345) **F**. Cell cycle changes were measured by cell cycle analysis using PI staining technique. *P <0.05, **P<0.01, ***P<0.001.

Previously, miR-675-5p has been reported to function as a novel tumor suppressor in non-small cell lung cancer (NSCLC). For example, down-regulation of miR-675-5p promotes cell growth, cell proliferation and colony formation in NSCLC [19]. However, the role of miR-675-5p in pancreatic cancer has not been studied. Thus, we first examined the relative expression of miR-675-5p among four pancreatic cancer cell lines (Patu8988, SW1990, Bxpc3 and Panc-1) by qRT-PCR assay. As shown in Figure 2A, SW1990 cells had the highest miR-675-5p expression while Patu8988 cells had the lowest miR-675-5p expression. Next, we determined the role of miR-675-5p in pancreatic cancer by miRNA mimics and miRNA inhibitors. As expected, Patu8988 cells transfected with miR-675-5p mimics revealed a significant higher level of miR-675-5p whereas SW1990 cells transfected with miR-675-5p inhibitors revealed a significant lower level of miR-675-5p (Figure 2B).

We used the Cell Counting Kit-8 (CCK-8) to detect the relative proliferation fold of the pancreatic cancer cells, and found that overexpression of miR-675-5p in Patu8988 cells could inhibit cell growth and proliferation while downregulation of miR-675-5p in SW1990 cells promoted cell growth and proliferation (Figure 2C). Furthermore, the colony formation assay showed that overexpression of miR-675-5p in Patu8988 cells formed tiny and scattered colonies when downregulation of miR-675-5p in SW1990 cells formed intensive and bulky colonies compared with controls (Figure 2D). These results suggest that miR-675-5p may be a tumor suppressor in pancreatic cancer.

To determine the potential mechanism underlying the inhibitory effect on cell growth by miR-675-5p, we performed Annexin V fluorescein isothiocyanate (V-FITC) apoptotic assay and cell cycle analysis by flow cytometry. Over-expression of miR-675-5p in Patu8988 cells can increase the rate of apoptosis (Figure 2E). However, down-regulation of miR-675-5p in SW1990 had no statistically significant change (Figure 2E) probably due to the low apoptosis rate of cancer cells. We also performed the cell cycle assay using PI staining technique. In Patu8988 cells the percentage S phase cells were decreased and G1 and G2 phase cells were increased. In SW1990 cells we did not observe significant cell cycle changes probably due to the high percentage of S phase cancer cells (Figure 2F). These results suggest that miR-675-5p may cause cell growth arrest and cell death.

### Mir-675-5p participates in caspase-3 related apoptosis signaling pathway

**Figure 3:**
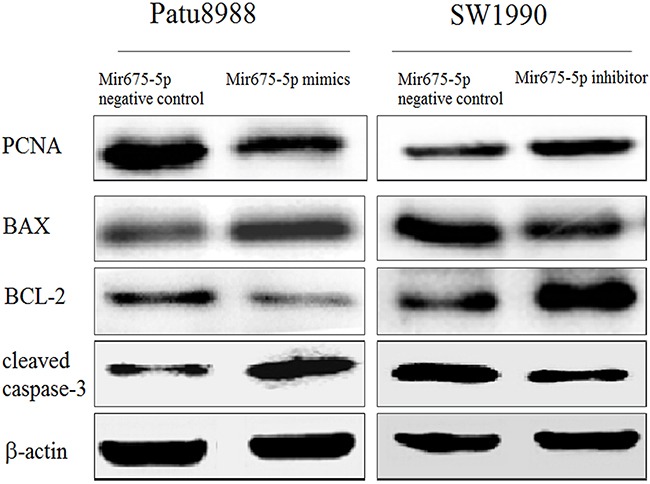
Mir-675-5p participates in caspase-3 related apoptosis signaling pathway Western blotting was used to analyze the PCNA, Bcl-2, Bax and cleaved Caspase-3 protein expressions in Patu8988 cells transfected with mir-675-5p mimics and SW1990 cells transfected with mir-675-5p inhibitor compared to negative controls.

The cellular function assays suggest that miR-675-5p impacts the proliferation and apoptosis of pancreatic cancer cells. To determine how miR-675-5p causes cell death and cell growth arrest, we examined a number of main apoptosis and proliferation related proteins, including PCNA, Bcl-2, Bax and cleaved caspase-3. In Patu8988 cells transfected with miR-675-5p mimics, PCNA and the ratio of Bcl-2 to Bax were decreased. On the other hand, the cleaved caspase-3 was activated. In SW1990 cells, miR-675-5p increased PCNA and the ratio of Bcl-2 to Bax and suppressed the cleaved caspase-3 level (Figure 3). The results support the important roles for miR-675-5p in proliferation and apoptosis in pancreatic cancer, possibly through caspase-3 related signaling pathway.

### Effect of mir-675-5p on cell migration and invasion of pancreatic cancer

**Figure 4:**
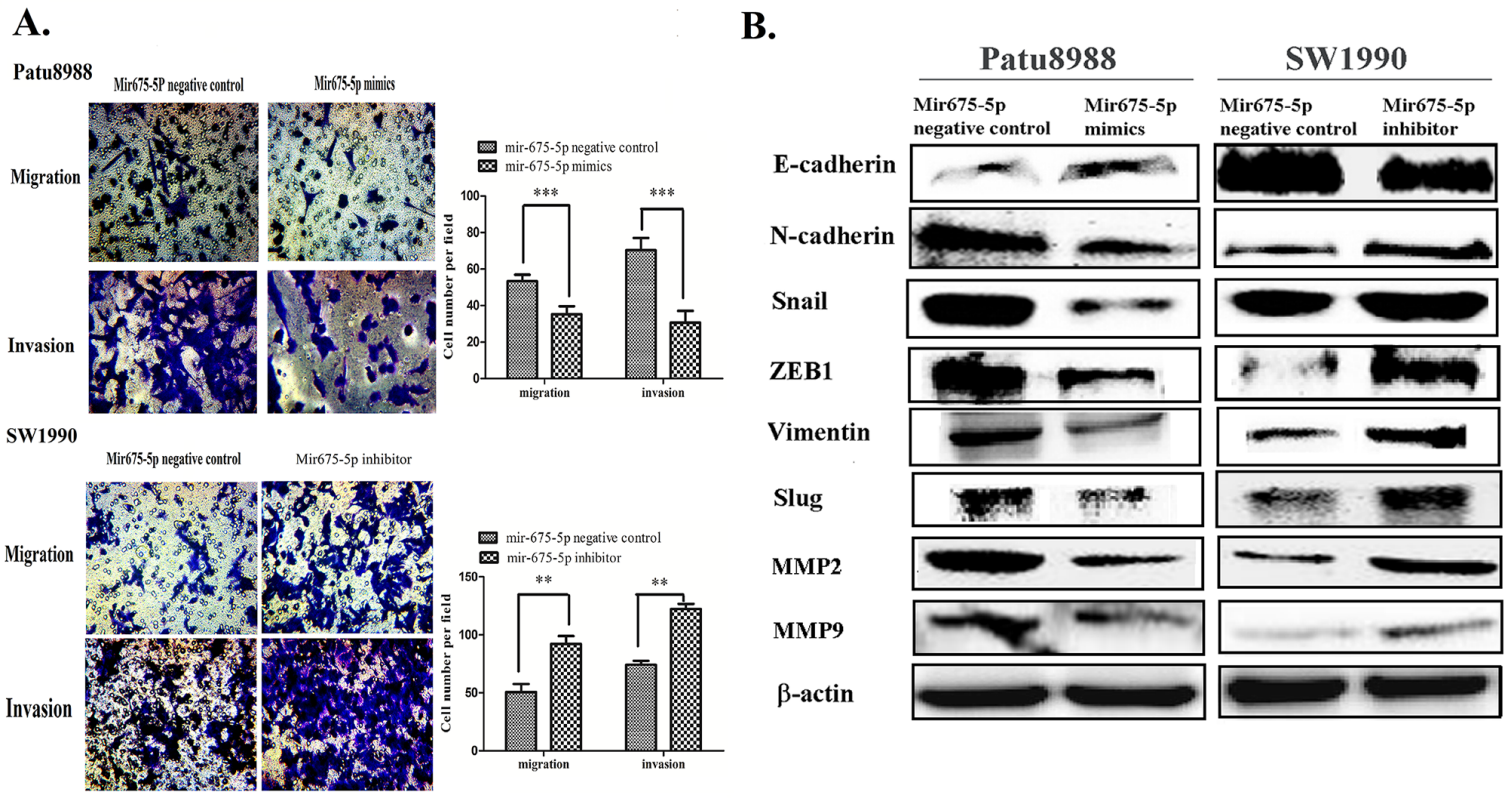
Mir-675-5p has an effect on cell migration and invasion of pancreatic cancer *in vitro* **A**. The abilities of cell migration and invasion were examined by transwell assay in Patu8988 cells transfected with mir-675-5p mimics and SW1990 cells transfected with mir-675-5p inhibitor. **B**. The protein levels of E-cadherin, N-cadherin, ZEB1, Vimentin, Snail, Slug, MMP2, MMP9 were detected by western blotting. The number of migration and invasion cells were calculated and depicted by the bar graph. **P<0.01, ***P<0.001.

Increased cell migration and invasion is often associated with EMT [20]. We detected the effect of miR-675-5p on cell migration and invasion by transwell assay. Upregulation of miR-675-5p in Patu8988 cells significantly impaired migration and invasion while downregulation of miR-675-5p promoted migration and invasion (Figure 4A). To further determine the potential role of miR-675-5p in the EMT signaling pathway, we examined a series of proteins related to EMT. In Patu8988 cells transfected with miR-675-5p mimics, the protein levels of E-cadherin were increased whereas the expression levels of N-cadherin, ZEB1, Vimentin, Snail and Slug were significantly decreased. In SW1990 cells transfected with miR-675-5p inhibitors, the levels of these proteins are in contrary to those for miR-675-5p mimics (Figure 4B).

Matrix metalloproteinase MMP2 and MMP9 are well known for their role in cell migration and invasion of cancer cells. We also detected that the protein levels of MMP2 and MMP9 were downregulated by miR-675-5p mimics in Patu8988 cells whereas miR-675-5p inhibitors upregulated MMP2 and MMP9 in SW1990 cells, suggesting that miR-675-5p can suppress the migration and invasion of pancreatic cancer.

### The role of UBQLN1-ZEB1-mir200 axis in the EMT of pancreatic cancer

**Figure 5:**
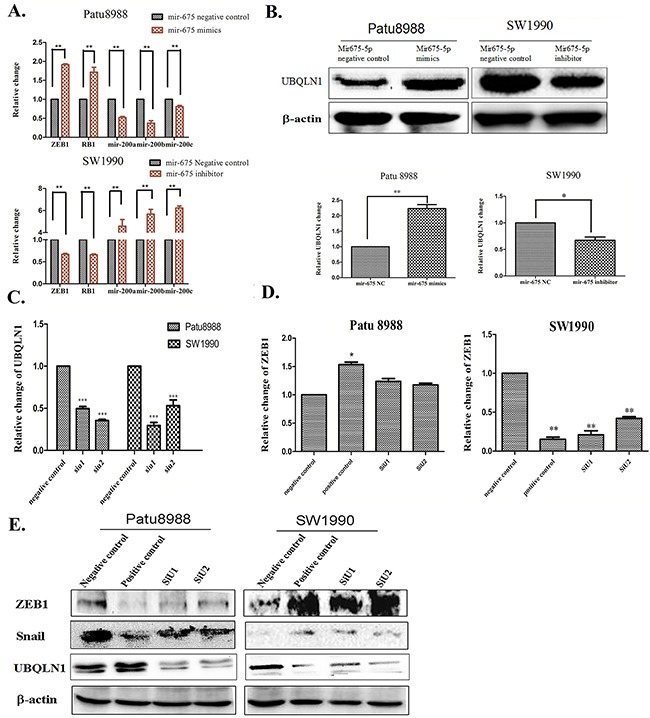
UBQLN1-ZEB1-mir200 axis plays a vital role in the EMT of pancreatic cancer **A**. The mRNA expression of ZEB1, RB1 and mir-200(a-c) were examined by qRT-PCR in Patu8988 cells transfected with mir-675-5p mimics and SW1990 cells transfected with mir-675-5p inhibitor. **B**. The protein levels of UBQLN1 were measured by western blotting and the mRNA levels were detected by qRT-PCR. **C**. The transfection efficiency of UBQLN1 siRNAs was detected by qRT-PCR. **D**. The mRNA expression of ZEB1 in Patu8988 cells cotransfected with mir-675-5p mimics and UBQLN1 siRNAs or SW1990 cells cotransfected with mir-675-5p inhibitor and UBQLN1 siRNAs was examined by qRT-PCR. **E**. The protein expressions of ZEB1, Snail and UBQLN1 were measured by western blotting in Patu8988 cells cotransfected with mir-675-5p mimics and UBQLN1 siRNAs or SW1990 cells cotransfected with mir-675-5p inhibitor and UBQLN1 siRNAs was examined by qRT-PCR. *P<0.05, **P<0.01, ***P<0.001.

Members of miR-200 family are usually considered as tumor suppressors and involved in EMT, and RB1 is confirmed as a direct target of miR-675 [21]. We examined the relative expression of miR-200a, miR-200b and miR-200c, ZEB1 and RB1 in Patu8988 and SW1990 cells, respectively, by qRT-PCR assay. In Patu8988 cells miR-675-5p mimics increased the relative expression of ZEB1 mRNA, but decreased miR-200a, miR-200b and miR-200c (Figure 5A). In contrast, miR-675-5p inhibitors decreased ZEB1 mRNA, but increased miR-200a, miR-200b and miR-200c in SW1990 cells (Figure 5A). The mir-675-5p can increase the expression of ZEB1 mRNA, but the ZEB1 protein level was decreased. It suggests that mir-675-5p may affect ZEB1 in a post-transcriptional level which was verified to be regulated by UBQLN1 protein.

It has been shown that there is a physiological, reciprocal regulation of EMT by UBQLN1 and ZEB1 [17]. Both the relative mRNA and protein levels of UBQLN1 were increased by miR-675-5p mimics in Patu8988 cells; in contrast, miR-675-5p inhibitors suppressed UBQLN1 in SW1990 cells (Figure 5B).

Finally, to determine whether miR-675-5p regulates the progression and development of pancreatic cancer via the UBQLN1-ZEB1-mir200 axis, we performed UBQLN1 knock down in Patu8988 cells transfected with miR-675-5p mimics and in SW1990 cells transfected with miR-675-5p inhibitor to detect changes in ZEB1 expression. We also included a negative control and a positive control for these cells. As shown in Figure 5C, UBQLN1 was significantly decreased by UBQLN1 siRNAs in both Patu8988 cells and SW1990 cells. Interestingly in Patu8988 cells, miR-675-5p mimics upregulated ZEB1 but this upregulation can be reversed by suppressing the expression of UBQLN1. In SW1990 cells, miR-675-5p inhibitors downregulated the mRNA level of ZEB1 and UBQLN1 siRNAs enhanced the results (Figure 5D). Western blot showed that the protein levels of ZEB1 and Snail were also decreased by UBQLN1 siRNAs in Patu8988 cells and the trend can be reversed by UBQLN1 siRNAs. In contrast, the protein levels of ZEB1 and Snail were increased by miR-675-5p inhibitors in SW1990 cells and the trend can be maintained by UBQLN1 siRNAs (Figure 5E). Thus, miR-675-5p can influence the expression of ZEB1 through UBQLN1.

## DISCUSSIONS

It is known that H19 and miR-675 may play a dual role in tumor progression and development [9]. They not only function as oncogenes in colorectal cancer [21], gastric cancer [22], and glioma [23], but also act as tumor suppressors in hepatocellular carcinoma [24]. However, most of these studies have focused on the role of H19 in cancer, but little work has been carried out on miR-675. As miR-675 is derived from the first exon of H19, it also plays an important role in tumor development in an H19 dependent manner [8].

Ma *et al* reported that H19 may play an oncogenic role in pancreatic cancer by increasing HMGA2-mediated EMT through antagonizing let-7 [25]. However, our study demonstrated that decreased expression of H19 had no effect on proliferation but significantly promoted the migration and invasion of pancreatic cancer cells (data not shown). Thus, we believe that H19 may act as a tumor suppressor in pancreatic cancer. These contradictory findings may be due to different cell lines we used. For example, we screened the expression of H19 in four pancreatic cancer cell lines and filtrated two cell lines (SW1990 and Bxpc3) which have high expression of H19 while two cell lines (Patu8988 and Panc-1) which have low expression of H19. Ma *et al* used H19 siRNA on Panc-1 cells which itself had low expression of H19 [25]. Our results are consistent with the report that H19 and miR-675 have higher expression in adjacent tissues compared to tumor tissues [11]. H19 and miR-675 may have a dual mechanism depending on the tumor microenvironment or tumor type. In this regard, H19 and its derived miR-675 may be tumor promoters in gastrointestinal cancers like gastric caner and colon cancer. On the other hand, they may play a tumor suppressive role in digestive gland tumors like pancreatic cancer and hepatocellular carcinoma.

The level of RB mRNA in Patu8988 cells is upregulated by miR-675-5p mimics while it is downregulated by miR-675-5p inhibitors in SW1990 cells. The results are consistent with the CCK-8 assays. RB is a direct target of miR-675 in colorectal cancer by incorporation into an RNA-induced silencing complex that binds to RB mRNA [21,26]. The expression of RB is supposed to be suppressed by miR-675-5p mimics, but our results fail to support this. It is possible that RB is a middle factor mediated by miR-675 or miR-675 which can stabilize RB mRNA.

ZEB proteins function as transcriptional repressors and ZEB1 has been shown to be direct suppressor of E-cadherin during EMT [17,27]. The mir-675-5p can increase the expression of ZEB1 mRNA, but the ZEB1 protein level was decreased. Our results support the finding that there is a reciprocal feedback loop between ZEB1 and miR-200 family [16]. The contradictory results suggest that miR-675 may regulate ZEB1 in a post-transcriptional level. UBQLN1 is a ubiquitin-like protein and can repress the expression of ZEB1; there is also a physiological, reciprocal regulation of EMT by UBQLN1 and ZEB1. The qRT-PCR and Western Blot results support the notion that UBQLN1 might be an intermediate factor in pancreatic caner which regulates ZEB1. Thus, we knock down UBQLN1 in Patu8988 cells transfected with miR-675-5p mimics and SW1990 cells transfected with miR-675-5p inhibitor to detect changes in ZEB1 expression. Importantly, miR-675-5p can influence the expression of ZEB1 by UBQLN1. We also detected the expression of ZEB1 and UBQLN1 in SW1990 cells treated with H19 siRNA (data not shown). Downregulation of H19 impaired the expression of ZEB1 mRNA and protein levels. The expression of UBQLN1 and miR-200 were not different as compared to the negative control. These results suggest that miR-675-5p can regulate the expression of UBQLN1 and miR-200 rather than H19 although miR-675 is embedded in the first exon of H19.

There was no direct relationship between miR-200(a-c) and miR-675, however, miR-200(a-c) expression changed depending on miR-675. For example, the relative expression of miR-200 decreased with miR-675 overexpression while it increased with miR-675 downregulation. The miR-200 and miR-675-5p may establish a relationship by ZEB1. Mir-200 had been commonly considered as tumor suppressor. However, miR-200a and miR-200b were reported to be hypomethylated and overexpressed in pancreatic cancer compared to adjacent mucosa [15]. Thus, miR-200 family cannot be simply regarded as oncogenes or tumor suppressors. Rather, they might reflect intracellular homeostasis and functional changes.

In summary, the miR-675-5p inhibits the development and progression of pancreatic cancer. Mir-675-5p could suppress the tumorigenesis of pancreatic cancer through inhibition of proliferation, migration, invasion and promotion of apoptosis. There is a correlation between miR-200 family and miR-675-5p which can be controlled by *ZEB1*. Mir-675-5p may affect ZEB1 in a post-transcriptional level which is also regulated by UBQLN1, leading MET.

## MATERIALS AND METHODS

### Cell lines and cell culture

Four cell lines (Patu8988, SW1990, Bxpc-3, and Panc-1) were obtained from Medical Science Institute of Jiangsu University. Cells were cultured in DMEM (Wisent) medium supplemented with 10% fetal bovine serum (Gibco) in humidified air at 37°C with 5% CO_2_.

### RNA extraction and qRT-PCR analyses

Total RNA was extracted from cell lines using Trizol reagent (Invitrogen) according to the manufacturer^’^s protocol. Levels of miR-675-5p and miR-200a, b and c were normalized to U6 and levels of ZEB1, UBQLN1 and RB were normalized to GAPDH, respectively to yield a 2^−DDCt^ value for relative expression of each transcript and a >35 Ct value indicated negative amplification. Experiments were repeated at least three times. The miRNA RT reaction was carried out under the following conditions: at 42°C for 60 min, at 70°C for 10 min. After the RT reaction, the complementary DNA products were diluted at 1:20 and 2ul of the diluted complementary DNA was used for subsequent qRT-PCR reactions. The miR-RNA primers were obtained from RIBOBIO in Guangzhou. Other qRT-PCR primers were listed in Table 1. The qRT-PCR reaction was conducted at 95°C for 20s and followed by 40 cycles of 95°C for 10s, 60°C for 20s and 72°C for 10s in the CFX 96 real-time PCR system (Applied Biosystems, CA, USA). The qRT-PCR results were analyzed and expressed as relative miRNA expression of Ct value, which was then converted to fold changes.

**Table 1:** The sequence of primers used in qRT-PCR

gene	Sequence (5′-3′)	Length (bp)
ZEB1	F: GGCAGAGAATGAGGGAGAAG	119
	R: CTTCAGACACTTGCTCACTACTC	
H19	F: TACAACCACTGCACTACCTG	575
	R: TGGAATGCTTGAAGGCTGCT	
RB1	F: GAAGCAGATGGAAGTAAACATCTCC	127
	R: CCTTGTTTGAGGTATCCATGCTATC	
UBQLN1	F: CTCATCCCAGGGTTTACTCC	196
	R: CTTCTGGATTCTGTAGCTGAGGA	

### Transfection of mircoRNA mimics and inhibitors

The hsa-miR-675-5p mimics, inhibitors (RIBOBIO Co, Guangzhou China) and the UBQLN1 siRNA (Genepharma) were transfected into the pancreatic cell lines for 48 h. 5 ul hsa-miR-675-5p mimics or inhibitors and lipofectamine 2000 (invitrogen, USA) were mixed up with 245 ul with opti-MEM(Gibco) respectively. 2.5 ul UBQLN1 siRNA and hsa-miR-675-5p mimics or inhibitors were mixed up with 245 ul opti-MEM(Gibco) when they were introduced into cells by co-transfection. Expression was validated by qRT-PCR. The UBQLN1 siRNA sequences were listed as follows:

UBQLN1 siRNA1: 5′-GAAGAAAUCUCUAAACGUU  UUUU-3′ (Sense)

         5′-AAAACGUUUAGAGAUUUCU  UCGG-3′ (Antisense)

UBQLN1 siRNA2: 5′-AACCUGGACAUCAGCAGU  UUA-3′ (Sense)

         5′-AACUGCUGAUGUCCAGGUU  CC-3′ (Antisense)

### Cell proliferation, cell cycle and colony formation assays

Cell proliferation was measured using cell counting Kit-8 (DoJindo, Japan). Mir-675-5p mimics or negative control was transfected to Patu8988 cells and miR-675-5p inhibitors or negative control was transfected to SW1990 (2000/well) and then allowed to grow in 96-well plates. Cell proliferation was documented every 24h following the manufacturer's protocol. Cell cycle analyses were performed using propidium iodide (Beyotime, China). For cell cycle analyses, cells were seeded in 6-well plates at 3 × 10^5^ per well. Forty-eight hours after transfection, cells were fixed in 70% ethanol at 4°C for 24h and stained with propidium iodide (Beytime, Beijing, China). The cell cycle distribution was analyzed by flow cytometry (BD FACSCalibur, American). For the colony formation assay, cells were allowed to grow in 6-well plates and maintained in media containing 10% FBS, replacing the medium every 4 days. After 10 days, cells were fixed with paraformaldehyde and stained with 0.1% crystal violet (Beytime, Beijing, China). Visible colonies were manually counted. The cell colony formation rate (%) was calculated by the formula: cell colony number/inoculating cell number ×100%. The inoculating cell number is 500, 1000, 1500 respectively. All experiments were performed in triplicates.

### *In vitro* cell migration and invasion assays

For the migration assays, 48 h after transfection, 7 × 10^4^ cells in 200ul serum-free media were placed into the upper chamber of an insert (8 um pore size, BD). The incubation time was about 14-16h. For the invasion assays, 1 × 10^5^ cells in 200ul serum-free media were placed into the upper chamber of an insert coated with Matrigel (BD, USA). The incubation time was about 36-40h. The medium containing 500ul FBS (10%) was added to the lower chamber. After incubation, the cells remaining on the upper membrane were removed with cotton wool, whereas the cells that had migrated or invaded through the membrane were fixed and stained with 0.05% crystal violet for 30 min. Cell number was counted using an inverted microscope (Canon, Japan) and repeated three times.

### Western blotting

Total protein was extracted by lysing cells in RIPA buffer containing protease inhibitors. Protein samples were separated by sodium dodecyl sulfate polyacrylamide gelelectrophoresis (SDS-PAGE) and transferred onto polyvinylidene fluoride (PVDF) membranes. After blocking with 5% non-fat milk in TBST, membranes were incubated with the primary antibody. Antibodies against ZEB1, cleaved caspased-3, E-cadherin, N-cadherin, Vimentin, Snail and Slug purchased from Cell Signaling Technology in USA were diluted at 1:1000. Bcl-2, Bax, PCNA, UBQLN1, MMP2, MMP9 and β-actin purchased from Proteintech Group in USA were diluted at 1:1000 and Peroxidase-conjugated Affinipure goat anti-mouse IgG (H+L) or anti-rabbit IgG (H+L) were diluted at 1:4000 as the secondary antibody.

### Flow cytometry

Samples were collected from 6-well plates, with cells being 70-90% confluent (0.5-1 × 10^6^ cells). Cells were harvested by trypsinization without EDTA after washing with 1x phosphate-buffered saline (PBS), and then centrifuged at 900 r/min for 4 min and resuspended in 1ml pre-cool PBS for two times. It was centrifuged at 750 r/min for 3 min and then it was resuspended with 1 ml binding buffer. 100 ul liquid was taken from the resuspension solution and 5 ul PI and Annexin V-FITC were added into it respectively. The assays were repeated three times.

### Statistics

Comparisons between groups were analyzed using the Student's t text (two groups) or a one-way ANVON (multiple groups). The survival curve was analyzed by Kaplan-Meier method using SPSS Statistics 20 and GraphPad Prism5. Differences with *P* values less than 0.05 were considered significant.
